# Discovery and Validation of a New Class of Small Molecule Toll-Like Receptor 4 (TLR4) Inhibitors

**DOI:** 10.1371/journal.pone.0065779

**Published:** 2013-06-12

**Authors:** Matthew D. Neal, Hongpeng Jia, Benjamin Eyer, Misty Good, Christopher J. Guerriero, Chhinder P. Sodhi, Amin Afrazi, Thomas Prindle, Congrong Ma, Maria Branca, John Ozolek, Jeffrey L. Brodsky, Peter Wipf, David J. Hackam

**Affiliations:** 1 Division of Pediatric Surgery Children’s Hospital of Pittsburgh and Department of Surgery, University of Pittsburgh School of Medicine, Pittsburgh, Pennsylvania, United States of America; 2 Department of Chemistry and Center for Chemical Methodologies and Library Development, University of Pittsburgh, Pittsburgh, Pennsylvania, United States of America; 3 Department of Biological Sciences, University of Pittsburgh, Pittsburgh, Pennsylvania, United States of America; 4 Division of Newborn Medicine, Children’s Hospital of Pittsburgh and Department of Pediatrics, University of Pittsburgh School of Medicine, Pittsburgh, Pennsylvania, United States of America; 5 Division of Pathology, Children’s Hospital of Pittsburgh and Department of Pathology, University of Pittsburgh School of Medicine, Pittsburgh, Pennsylvania, United States of America; Louisiana State University, United States of America

## Abstract

Many inflammatory diseases may be linked to pathologically elevated signaling via the receptor for lipopolysaccharide (LPS), toll-like receptor 4 (TLR4). There has thus been great interest in the discovery of TLR4 inhibitors as potential anti-inflammatory agents. Recently, the structure of TLR4 bound to the inhibitor E5564 was solved, raising the possibility that novel TLR4 inhibitors that target the E5564-binding domain could be designed. We utilized a similarity search algorithm in conjunction with a limited screening approach of small molecule libraries to identify compounds that bind to the E5564 site and inhibit TLR4. Our lead compound, C34, is a 2-acetamidopyranoside (MW 389) with the formula C_17_H_27_NO_9,_ which inhibited TLR4 in enterocytes and macrophages *in vitro,* and reduced systemic inflammation in mouse models of endotoxemia and necrotizing enterocolitis. Molecular docking of C34 to the hydrophobic internal pocket of the TLR4 co-receptor MD-2 demonstrated a tight fit, embedding the pyran ring deep inside the pocket. Strikingly, C34 inhibited LPS signaling *ex-vivo* in human ileum that was resected from infants with necrotizing enterocolitis. These findings identify C34 and the β-anomeric cyclohexyl analog C35 as novel leads for small molecule TLR4 inhibitors that have potential therapeutic benefit for TLR4-mediated inflammatory diseases.

## Introduction

The innate immune receptor toll-like receptor 4 (TLR4) has been recognized as the receptor on hematopoietic and non-hematopoietic cells for bacterial endotoxin (lipopolysaccharide, “LPS”) [1], as well as for a variety of endogenous molecules that are released during inflammatory or infectious disorders [2]. A number of diseases have been attributed to exaggerated TLR4 signaling, including both infectious and non-infectious processes. These include necrotizing enterocolitis (NEC) [3], abdominal sepsis [4], pneumonia [5], arthritis [6], pancreatitis [7] and atherosclerosis [8]. Strategies to discover molecules that can neutralize TLR4 signaling are thus predicted to show great promise as novel anti-infective and/or anti-inflammatory agents.

The discovery of agents with anti-TLR4 properties has so far been met with limited success, which until recently could be attributed in part to a lack of reliable structural information on the LPS signaling site on TLR4. Prior strategies to prevent LPS signaling have therefore focused on the molecule LPS itself, which is known to contain three distinct domains, including lipid A (the bioactive component that is recognized in causing human infection), a short oligosaccharide core, and the O-antigen polysaccharide that varies in composition amongst gram-negative bacterial strains [9]. The elucidation of the structure of LPS led to the identification of the synthetic lipid A analogue eritoran (E5564), as well as the lipid A mimetic CRX-526 in which the reducing sugar on lipid A was replaced with an *N*-acylated aminoalkyl saccharide [10]. Both E5564 and CRX-526 inhibited LPS signaling, but, unfortunately, lacked efficacy in clinical trials [11], [12].

Recently, the crystal structure of the TLR4-MD2 complex with bound E5564 was solved, which suggested that the mechanism of action of E5564 involves its association with a large internal pocket in the TLR4 co-receptor MD-2 [13]. This structural information provides new opportunities for the design of novel E5564 analogs with the ability to inhibit TLR4 signaling. We now report the identification of a new class of small organic molecules with anti-TLR4 properties and reveal the potential of our lead compound to serve as an anti-inflammatory agent in diseases characterized by increased TLR4 signaling.

## Materials and Methods

### Assessment of TLR4 Inhibition in Mice

All mice were housed and cared for at the Rangos Research Center of the Children’s Hospital of Pittsburgh (Pittsburgh, PA), and all experiments were approved by the Institutional Review Board of the University of Pittsburgh (Permit number: 12040382). All studies were carried out in strict accordance with the recommendations in the Guide for the Care and Use of Laboratory Animals of the National Institutes of Health. Throughout the course of all experiments, mice were housed with access to food, water and standard bedding. Endotoxemia was induced in all experiments by intraperitoneal injection of LPS (*Escherichia coli* 0111:B4 purified by gel filtration chromatography, >99% pure, Sigma-Aldrich) at a dose of 3 mg/kg for 6 hours into 6 week old male mice. At the end of each experiment, all animals were euthanized by CO_2_ and cervical dislocation. Immediately prior to injection into mice, the compounds were diluted to an experimental concentration of 100 uM in PBS, with the total concentration of DMSO in the final diluted drug at 1%. Compounds were closely examined to insure that no precipitate formed prior to injection and were stored on ice until injection. In all experiments listed, compounds were delivered to 6 week old mice 30 minutes prior to injection with LPS. Control animals not receiving compound received 1% DMSO dissolved in PBS (“vehicle controls”). Where indicated, mice were also injected with LPS along with the NFκB inhibitor Bay-11-7082 (20 mg/kg, 30 min pretreatment i.p., Cayman Chemical). In addition to assessing the effect on clinical activity of the mice in which the degree of piloerection, tachypnea and movement activity (huddled in the corner versus roaming freely) were assessed, LPS and individual compounds were also injected into NFκB-luciferase reporter mice, in which NFκB is upstream of the luciferase gene (strain NFκB-RE-luc, Taconic Farms Inc, Hudson, NY). In these studies, 6h after LPS injection, mice were administered an i.p. injection of luciferin (160 ug/kg, Caliper Life Sciences), then after 10 minutes, a whole animal image to evaluate luciferase activity was obtained using the IVIS Lumina 3D Optical in vivo imaging system (Caliper Life Sciences, Hopkinton, MA) under 1.5% isofluorane anesthesia. Prior to being euthanized, mice from the above experiments were anesthetized with 1.5% isofluorane and a retro-orbital sinus puncture was performed to obtain a blood sample; serum was obtained via centrifugation and ELISA was performed to assess IL-6 expression (R&D Biosystems). The extent of expression of the pro-inflammatory cytokines IL-6 and iNOS within the intestinal mucosa was determined by RT-PCR (see below).

### In vitro Determination of TLR4 Inhibition

The ability of the individual compounds to inhibit TLR4 *in vitro* was determined in cultured enterocytes (non-transformed rat small intestinal IEC-6 cells) and monocytes (mouse RAW 264.7 cells). Both IEC-6 cells and RAW 264.7 cells were obtained from the American Type Culture Collection (ATCC, Manassas, VA). Cells were treated with individual compounds at a concentration of 10 uM 30 min prior to treatment with LPS (LPS dose was 10 ng/ml in RAW 264.7 cells, 10 ug/ml in IEC-6 cells), and the extent of LPS signaling was determined by the degree of TNFα expression by qRT-PCR. In parallel, RAW 264.7 cells were transduced with an adenovirus expressing the NFκB-luciferase reporter gene – a kind gift from Dr. Paul McCray, University of Iowa, as described [14], and then treated with LPS at 10 ng/ml after pre-treatment with the individual compounds at 100 µM. The NFκB-luciferase activity was measured using the Luciferase Activity System (Promega, Madison, WI) according to the manufacturer’s directions, and relative light units were normalized by total protein content in each sample tested.

### Synthesis of C34 and C35 and Preparation of Labeled ^3^H-C35

The route for the synthesis of C34 that was used to validate the structure of the commercial sample of this compound, as well as the preparation of analog C35 and its tritiated derivative, ^3^H-C35 is described in detail below (see Results). A synthetic access to C34 was necessary since the commercial sample was of unknown relative and absolute configuration. Spectral data for a validated sample of C34∶^1^H NMR (400 MHz, CDCl_3_) δ 5.62 (d, *J* = 9.6 Hz, 1 H), 5.19 (app t, *J* = 9.6 Hz, 1 H), 5.10 (app t, *J* = 9.6 Hz, 1 H), 4.92 (d, *J* = 3.8 Hz, 1 H), 4.30 (td, *J* = 3.6, 9.6 Hz, 1 H), 4.22 (dd, *J* = 4.8, 12.4 Hz, 1 H), 4.08 (dd, *J* = 2.4, 12.4 Hz, 1 H), 4.03–3.99 (m, 1 H), 3.88 (sept, *J* = 6.0 Hz, 1 H), 2.07 (s, 3 H), 2.02 (s, 3 H), 2.01 (s, 3 H), 1.93 (s, 3 H), 1.23 (d, *J* = 6.4 Hz, 3 H), 1.14 (d, *J* = 6.4 Hz, 3 H); ^13^C NMR (100 MHz, CDCl_3_) δ 171.4, 170.7, 169.8, 169.3, 95.7, 71.4, 71.0, 68.2, 67.7, 62.0, 51.8, 23.2, 23.1, 21.6, 20.7 (2 C), 20.6; HRMS (ESI) *m/z* calcd for C_17_H_28_NO_9_ (M+H) 390.1764, found 390.1790. A *de novo* synthesis was also required to prepare a tritiated derivative of C35, which was selected for labeling due to the equivalent bioactivity of C34 and C35, and the relative ease to introduce tritium by tritiation of the alkene precursor **4**. In brief, a solution of **4** (0.4 mg, 0.001 mmol) in anhydrous ethyl acetate (0.2 mL, 0.005 M) and 10% Pd/C (1 mg) was cooled to −78°C in a dry ice bath, evacuated at −78°C and pressurized to 635 Torr with tritium gas in a Trisorber loaded with 45 Ci of tritium. The dry ice bath was removed, allowing the pressure to rise to 800 Torr. Residual tritium gas outside the reaction chamber was reabsorbed in the Trisorber. After 4 h, the reaction mixture was cooled to −78°C and tritium gas was reabsorbed until the pressure fell to 52 Torr. The mixture was then stirred at room temperature overnight, allowing the pressure to rise to 130 Torr. Nitrogen was used to increase the internal pressure to 760 Torr before the reaction vessel was removed from the Trisorber. The mixture was treated with 0.8 mL of ethyl acetate, filtered through a pad of celite and glasswool in a Pasteur pipette and rinsed with 0.5 mL of ethyl acetate. A total of 1.5 mL of solvent was collected in a 1.8 mL screw top vial ( = “parent solution”). Spectral data for C35 [15]: ^1^H NMR (400 MHz, CDCl_3_) δ 5.57 (d, *J* = 8.4 Hz, 1 H), 5.40 (app. t, *J* = 9.2 Hz, 1 H), 5.04 (t, *J* = 9.6 Hz, 1 H), 4.86 (d, *J* = 8.4 Hz, 1 H), 4.26 (dd, *J* = 4.8, 12.0 Hz, 1 H), 4.10 (dd, *J* = 2.4, 12.0 Hz, 1 H), 3.72–3.58 (m, 3 H), 2.07 (s, 3 H), 2.02 (s, 3 H), 2.01 (s, 3 H), 1.93 (s, 3 H), 1.91–1.18 (m, 10 H); ^13^C NMR (100 MHz, CDCl_3_) δ 170.8, 170.2, 169.5, 98.9, 77.7, 72.1, 71.5, 68.9, 62.3, 55.5, 33.2, 31.6, 25.5, 23.8, 23.7, 23.4, 20.8, 20.7 (2 C).

An aliquot of 10 uL was removed, diluted in 1 mL of ethyl acetate, and 10 uL of this mixture was analyzed in a Packard scintillation counter, giving 4,500,000 cpm, equivalent to 90,000,000,000 dpm/mL of the original solution. Assuming 2,220,000,000 dpm/mCi, the original solution therefore has an activity of approximately 40 mCi/mL, or a total of 60 mCi (ca. 150 mCi/mg) of ^3^H-C35, with an experimental specific activity of 60 mCi/umol. Of note, the calculated maximum specific activity for ^3^H-C35 is 57.6 Ci/mmol, i.e. 57.6 mCi/umol, or 135 uCi/ug, in excellent agreement with the measured value.

A sample of 0.25 mL of the parent solution was transferred into a separate 1.8 mL screw top vial and evaporated under a nitrogen stream to give ^3^H-C35 (estimated to be approximately 0.07 mg) as a colorless residue. Based on the calculations of the parent solution, this sample is estimated to contain slightly less than 10 mCi activity.

### In vitro Assay to Measure TLR4 Binding

Wild type and TLR4 knockout bone marrow-derived macrophages (generous gift of Dr. Russ Salter, University of Pittsburgh) were seeded onto 6 well plates and allowed to reach confluence, which took ∼2–3 days. The plates were chilled on ice and the cells were washed four times with ice cold Hanks balanced salt solution (HBSS) supplemented with 0.6% BSA. Prior to this time, a total of 2 nmol of ^3^H-C35, prepared as described above, was mixed with 2 mmol of unlabeled C35 in a total volume of 1 mL of HBSS supplemented with 0.6% BSA. The unlabeled material enhanced compound binding (data not shown) since the apparent K_0.5_ for the cellular effects of C35 is relatively high (see text). Next, the solution containing C35 was added onto the cells and incubated for 1 hr on ice. The cells were then washed four times as before and lysed in 1 ml of a solution containing 50 mM Tris pH 8, 100 mM EDTA, 0.4% sodium deoxycholate, and 1% NP-40 for 5 min on ice. The lysates were transferred to scintillation vials containing 2 ml of ScintiSafe (ThermoFisher) and analyzed in a Beckman LS6000IC scintillation counter.

### Animal and Human Necrotizing Enterocolitis (NEC)

NEC was induced in neonatal mouse pups using our established model as previously reported through the combination of the administration of enteric formula and hypoxia in a manner that results in patchy necrosis of the small and large intestine that closely resembles clinical NEC. In brief, experimental NEC was induced in 7–8 day old mice as we have previously described [16] using formula gavage (Similac Advance infant formula (Abbott Nutrition):Esbilac canine milk replacer 2∶1) five times/day, and hypoxia (5% O_2_, 95% N_2_) for 10 minutes in a hypoxic chamber (Billups-Rothenberg) twice daily for 4 days. C34 was then administered via the oral route at a concentration of 1 mg/kg on each morning of the four day model. The severity of disease was determined on histologic sections of the terminal ileum by a pediatric pathologist who was blinded to the study condition according to our previously published scoring system from 0 (normal) to 3 (severe) [17]. Sections of the terminal ileum were harvested at the end of the model and processed for RNA, protein, and immunopathology analysis [18].

Human tissue was obtained after written, informed consent was received from the families of infants undergoing intestinal resection for active NEC after the nature and possible consequences of the studies were explained. The study was performed under an active research protocol that had been approved by the Institutional Review Board (IRB) of the University of Pittsburgh (number PRO09110437), which has jurisdiction over all research performed at the Children’s Hospital of Pittsburgh of the University of Pittsburgh Medical Center (UPMC) which is the site from which all the patient material was obtained.

After initial review by the duty pathologist to ensure that adequate diagnostic information was obtained from the gross tissue specimen, the intestinal resection was divided into multiple pieces, and treated with either LPS 100 ng/ml, C34 alone (10 uM) or LPS+ C34. After 3h, tissue was processed for RT-PCR, and assessed for the expression of TNFα and iNOS. Data from separate tissue harvests under each treatment group was then pooled and statistical analysis was performed.

### RT-PCR

Total RNA was isolated from cultured enterocytes or ileal mucosal scrapings from control and NEC mouse and human intestine using the RNeasy kit (Qiagen), and reverse transcribed (1 µg of RNA) using the QuantiTect Reverse Transcription Kit (Qiagen). Quantitative real-time PCR was performed using the ddCt method GAPDH as a housekeeping gene as we have previously described [3], [19]–[21] using the Bio-Rad CFX96 Real-Time System (Biorad, Hercules, CA) using the primers described in **Table 1**.

**Table 1:** Primers used in the current study.

Gene	Forward primer	Reverse Primer
Mouse GAPDH	TGAAGCAGGCATCTGAGGG	CGAAGGTGGAAGAGTGGGAG
Human GAPDH	TCTCCTCTGACTTCAACAGCGACA	CCCTGTTGCTGTAGCCAAATTCGT
Mouse IL6	CCAATTTCCAATGCTCTCCT	ACCACAGTGAGGAATGTCCA
Human IL6	TCTCCACAAGCGCCTTCG	CTCAGGGCTGAGATGCCG
Mouse TNFα	TTCCGAATTCACTGGAGCCTCGAA	TGCACCTCAGGGAAGAATCTGGAA
Human TNFα	GGCGTGGAGCTGAGAGATAAC	GGTGTGGGTGAGGAGCACA
Mouse iNOS	CTGCTGGTGGTGACAAGCACATTT	ATGTCATGAGCAAAGGCGCAGAAC
Human iNOS	AATGAGTCCCCGCAGCCCCT	AGTCATCCCGCTGCCCCAGT

### Statistical Analysis

All experiments were repeated at least in triplicate, with more than 100 cells/high-power field. For endotoxemia, at least 3 mice/group were assessed; for NEC, over 10 mouse pups per group were included and litter matched controls were included in all cases. Statistical analysis was performed using SPSS 13.0 software. ANOVA was used for comparisons for experiments involving more than two experimental groups. Two-tailed student’s t-test was used for comparison for experiments consisting of two experimental groups. For analysis of the severity of NEC, chi-square analysis was performed. In all cases, statistical significance was accepted at p<0.05 between groups.

## Results

### Selection of Potential TLR4 Inhibitors and Screening Paradigm

In order to identify new TLR4 inhibitors, we performed *in silico* similarity searches based on E5564 using the iResearch System Library of ChemNavigator®, the substance similarity Tanimoto scores implemented in SciFinder®’s substructure module, and ChemAxon®’s JChem® tools. From an initial yield of 700 hits, ∼10% (68 individual compounds) were cherry picked based on their commercial availability and chemical diversity. All members of this subset were then evaluated for their ability to inhibit TLR4 signaling in mice after LPS injection, and confirmed *in vitro* for their ability to inhibit LPS-induced TLR4 activation in both hematopoietic and epithelial cells. One compound, C34, was identified to meet or exceed our criteria of synthetic tractability, assay robustness, potency, and selectivity. While a few other hits were similarly potent, C34 had superior physicochemical properties. In particular, C34 has an attractive cLogP of −0.5, a topological polar surface area (TPSA) of 126, which is well with drug-like compound space, and passes both the Lipinski rules and the Veber filter [22]. Data on the remaining compounds is not included in the current manuscript. The parent compound C34 and the β-anomeric cyclohexyl analog C35, as well as a tritium-labeled derivative, ^3^H-C35, were synthesized to confirm the structural assignment, to establish a structure-activity relationship, and to begin to characterize its mechanism of action.

### In vivo Verification of TLR4 Inhibition by C34

**Figure 1:**
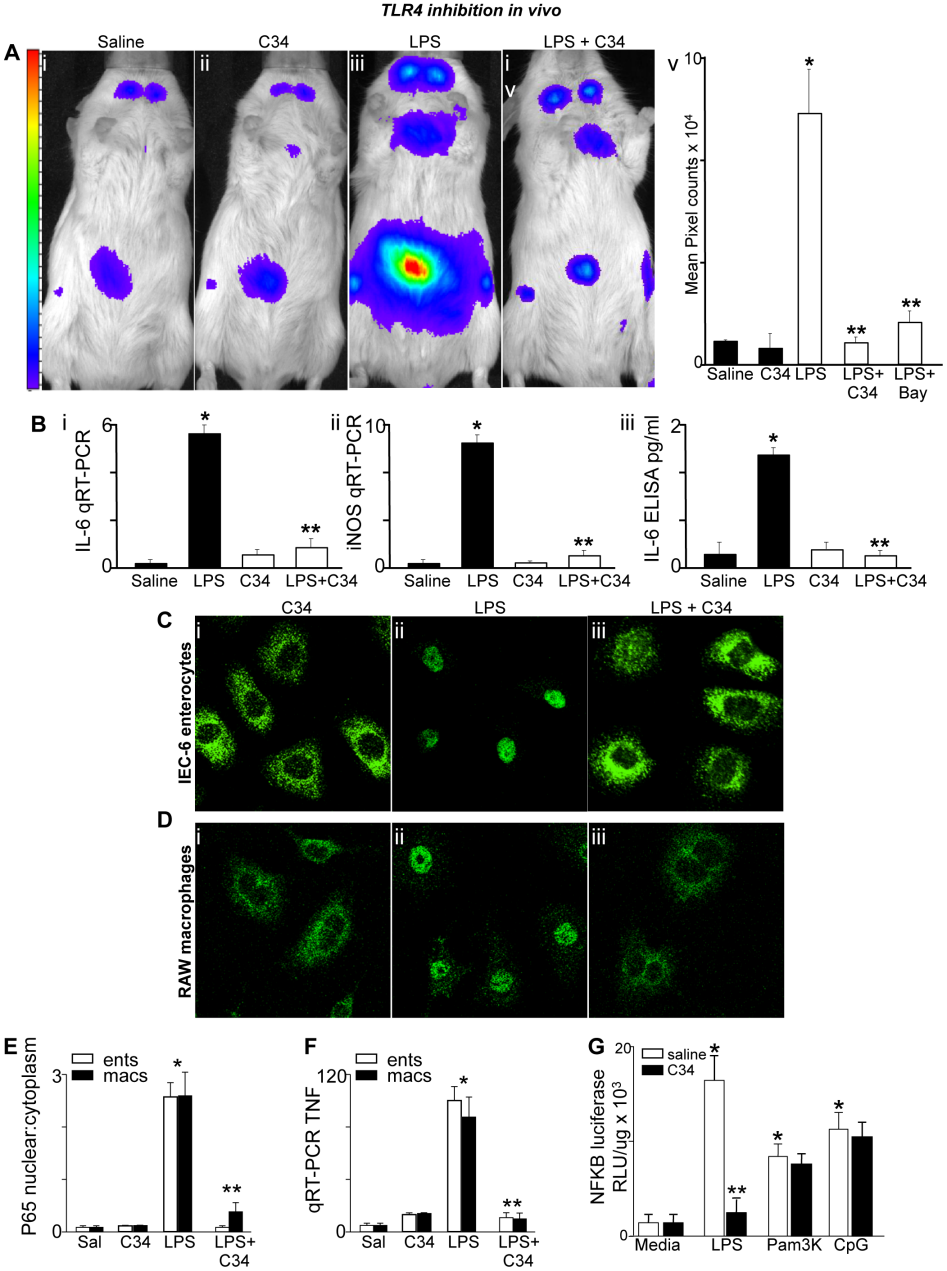
The novel inhibitor C34 blocks TLR4 signaling in vitro and in vivo. **A**: Pseudocolor images showing whole animal emission corresponding to NFκB-luciferase activity in mice that were treated with either saline (**i**), C34 (1 mg/kg) (**ii**), LPS (3 mg/kg, **iii**), and LPS after the pre-treatment with C34 (1 mg/kg, **iv**) 30 minutes prior; quantification (mean±SEM) is shown in **v** in which *p<0.05 LPS versus saline or C34 alone; **p<0.005 LPS vs LPS+C34 or LPS+Bay11 (20 mg/kg); summary of 4 separate experiments with over 5 mice per group. **B**: qRT-PCR for IL-6 (**i**) and iNOS (**ii**) in the small intestinal mucosa and IL-6 ELISA (**iii**) from the serum obtained from mice that were injected with either saline, LPS (3 mg/kg), C34 (1 mg/kg) or LPS 30 minutes after pre-treatment with C34. In each graph, data are mean±SEM in which *p<0.05 LPS vs saline; **p<0.05 LPS vs LPS+C34. Representative of 4 separate experiments with over 5 mice per group. **C–D**: Representative confocal micrographs showing the staining of p65-NFκB subunit in either IEC-6 cells (C) or RAW 264.7 macrophages (D) that were treated with C34 (**i**, 10 uM), LPS (**ii,** 10 ug/mL for IEC-6, 10 ng/mL for RAW 264.7), or LPS+C34 (**iii**). **E**: Quantification (mean±SEM) of NFκB translocation corresponding to experiments shown in Panels C and D for IEC-6 (white bars) and RAW 264.7 (black bars) under the conditions indicated. **F**: qRT-PCR (mean±SEM) of TNFα in IEC-6 (white) and RAW (black) cells under the conditions indicated; for **E** and **F**: *p<0.05 LPS vs saline or C34; **p<0.05 LPS vs LPS +C34 alone; **G**: luciferase activity in NFκB-luciferase transformed RAW 264.7 cells that were treated with either saline (white bars) or C34 (black bars) then with either media alone or LPS, Pam3K or CpG-DNA as indicated. Representative of 3 separate experiments; shown are mean±SEM. *p<0.05 LPS vs media; **p<0.05 LPS+saline vs LPS+C34 (C34 did not inhibit either Pam3K or CpG-DNA treated cells). C34 was administered both in vivo and in vitro 30 minutes prior to LPS in all cases. The y axes in panels B and F indicate the fold increase of the indicated gene relative to the housekeeping gene GAPDH.

Evidence in support of the ability of C34 to inhibit TLR4 signaling *in vivo* and *in vitro* is shown in **Figure 1**. To investigate the degree to which C34 inhibits LPS signaling *in vivo*, we injected LPS in the presence or absence of C34 into the NFκB-luciferase transgenic mouse strain, in which the luciferase gene is placed downstream of the TLR4-dependent transcription factor NFκB [23]. LPS signaling was then assessed through measurements of whole animal luciferase activity. As shown in **Figure 1A**, LPS injection caused a marked increase in luciferase activity – represented in pseudocolor format – which reveals the majority of the luciferase signal localized to the intestines and the lymph nodes within the neck. Pre-injection of mice with C34 30 minutes prior significantly decreased luciferase (i.e., TLR4-NFκB) activity in mice after LPS injection (**Figure 1A** **iii** versus **iv** and **v**). In control experiments, pre-treatment with the NFκB inhibitor bay-11 had a protective effect on the extent of LPS signaling as measured by a reduction in the extent of luciferase activity that was comparable to the effects of C34. Pre-treatment with C34 30 minutes prior also markedly reduced the extent of LPS-induced IL-6 and iNOS expression in the intestinal mucosa as detected by qRT-PCR (**Figure 1B** **i** and **ii**), and significantly reduced the extent of IL-6 release into the serum as assessed by ELISA (**Figure 1B** **iii**).

To determine whether C34 inhibits TLR4 signaling *in vitro,* we treated IEC-6 enterocytes and RAW 264.7 macrophages with LPS in the presence of C34 or vehicle control. LPS exposure activated TLR4 signaling in macrophages and enterocytes, as manifested by an increase in NFκB translocation from the cytoplasm to the nucleus (**Figure 1C–E** **)**, and an increase in the expression of TNFα by RT-PCR (**Figure 1F** **)**. Each of these events was significantly reduced by pretreatment with C34 when administered 30 minutes prior.

To evaluate whether the effects of C34 were specific for TLR4 agonists or whether C34 generally inhibited other TLRs, we treated RAW 264.7 cells that were stably transduced with the NFκB-luciferase gene with agonists for TLR4, TLR2 (Pam3K) or TLR9 (CpG-DNA) in the presence or absence of C34. As shown in **Figure 1G**, C34 only inhibited signaling via TLR4, and had no statistically significant effect on signaling via any other TLR receptor.

### C34 Docks into the Hydrophobic Pocket in MD-2

**Figure 2:**
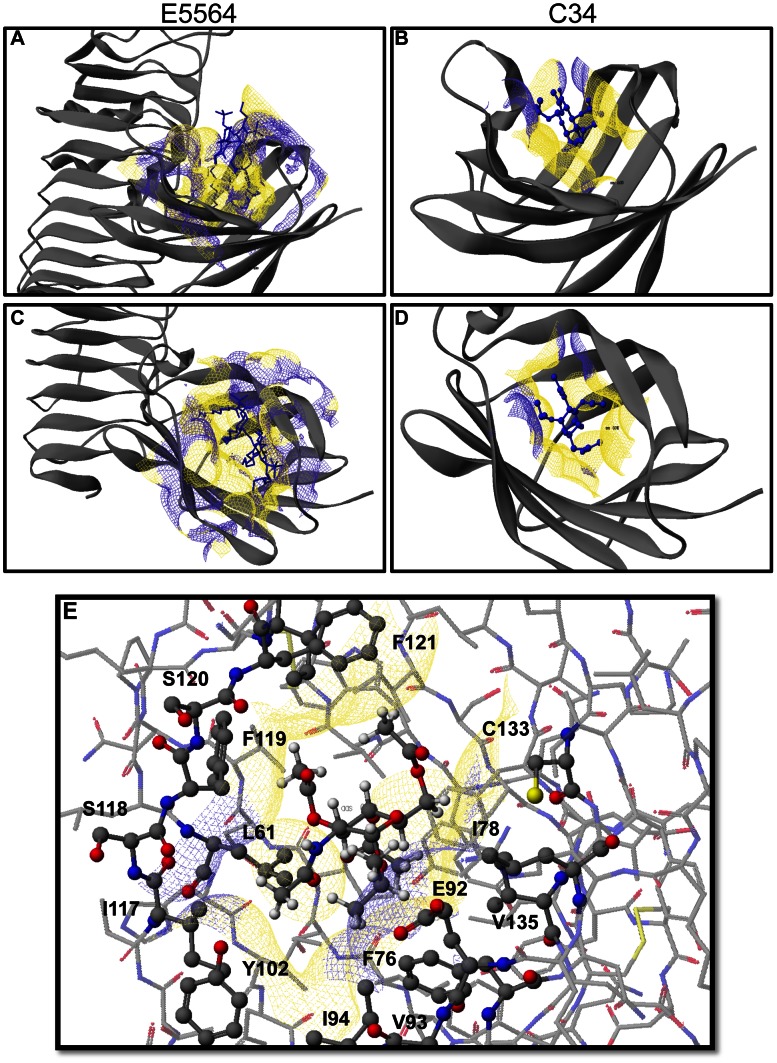
Molecular interactions of C34 with the TLR4-MD2 complex. X-ray structure of E5564 (**A** and **C**) and docking model of C34 (**B** and **D**) with the TLR4-MD2 complex, illustrating the interaction of E5563 and C34, respectively, with the hydrophobic groove of MD-2. Panel **E** displays the amino acid residues on hMD2 within a 5 Å contact radius of C34. See Results for additional details.

Based on the crystal structure of the TLR4-MD-2 complex with E5564 [13], we performed an *in silico* docking study with C34. The compound was first minimized using the MM2 force field in Scigress 7.7, and then superimposed on antagonist E5564. A genetic algorithm using a potential of mean force (PMF) and a maximum generation number of 3,000 was used to optimize the binding mode. As shown in **Figure 2**, C34 slides deeper into the hydrophobic grove of MD-2 than the disaccharide unit of E5564 (**Figure 2B and D** for C34 versus **A** and **C** for E5564), covering some of the space that is otherwise occupied by the four acyl chains of the endotoxin. There is sufficient room to accommodate both α- and β-anomers of C34, as well as larger hydrophobic anomeric substituents than *i*-propyl, which is in agreement with the structure-activity relationships of this small molecule inhibitor. The binding site for C34 is lined with mainly hydrophobic amino acid side chains, including F121, F119, L61, I117, Y102, I94, V93, F76, V135, and I78, with the notable exception of E92. The latter residue is in close vicinity of the anomeric carbon of C34, pointing its two methylene groups toward the isopropyl substituent of C34 and turning the carboxylate into the solvent space (**Figure 2E**).

### The C34 Analog C35 Inhibits TLR4 via Direct Binding

**Figure 3:**
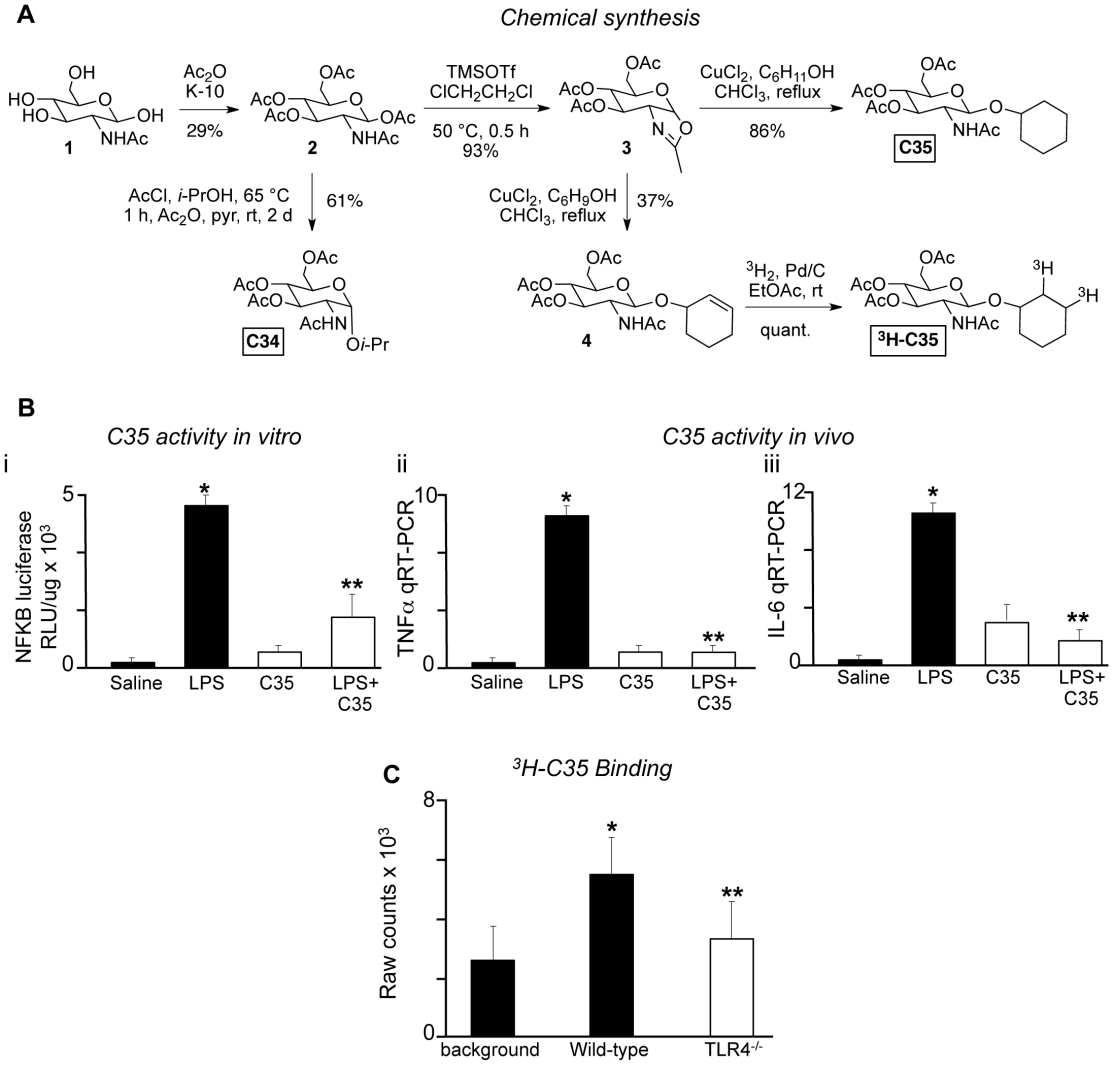
The C34 analog C35 inhibits TLR4 via direct binding. **A**: Synthesis of C34, its β-anomeric cyclohexyl analog C35 as well as the corresponding tritiated derivative (^3^H-C35). **B**: **i:** Luciferase activity in NFκB-luciferase-transduced RAW 264.7 macrophages exposed to saline, LPS (10 ng/ml), C35 (100 uM) or LPS 30 minutes after treatment with C35 as indicated. **ii–iii**: qRT-PCR showing the expression of TNFα (**ii**) and IL-6 (**iii**) in the small intestinal mucosa of mice that were injected with saline, LPS (3 mg/kg), C35 (1 mg/kg) or LPS 30 minutes after treatment with C35. In panels **i–iii**, *p<0.05 LPS vs saline treated mice or cells; **p<0.05 LPS vs LPS+C35 vs treated mice or cells. Representative of 3 separate experiments with at least 3 mice per group. Data are mean±SEM. **C:** Raw radiation counts×10^3^ from macrophages obtained from either wild-type or TLR4^−/−^ mice that had been treated with 50 µCi of ^3^H-C35, as described in Methods. The background emission is shown. Data are mean±SEM from 3 individual experiments. *p<0.05 wild-type vs background; **p<0.05 TLR4^−/−^ versus wild-type.

To ascertain whether C34 binds to TLR4 directly, the β-anomeric cyclohexyl analog C35 as well as the corresponding tritiated derivative (^3^H-C35) were synthesized as shown in **Figure 3A**. We then established that the unlabeled C35 compound significantly inhibited TLR4 signaling *in vitro*, as measured by a loss of LPS-induced NFκB-luciferase activity in RAW 264.7 cells (**Figure 3B** **i**), and *in vivo,* as measured by reductions in LPS-induced expression of the pro-inflammatory cytokines TNFα (**Figure 3B** **ii**) and IL-6 (**Figure 3B** **iii**) in the intestinal mucosa of mice that had been injected with LPS in the presence or absence of C35. To assess whether C35 directly interacted with TLR4, we seeded wild type and TLR4-knockout bone marrow-derived macrophages onto 6 well plates, and incubated these cells with unlabeled C35 as well as tritiated ^3^H-C35. As shown in **Figure 3C,** ^3^H-C35 bound RAW 264.7 macrophages that expressed TLR4 ∼1.5-fold better than TLR4-deficient cells. Taken together, the data presented in Figures 1 and 3 strongly suggest that both C34 and C35 interact directly with TLR4.

### C34 Attenuates the Severity of Experimental NEC, a Disease that Results from Exaggerated TLR4 Signaling

**Figure 4:**
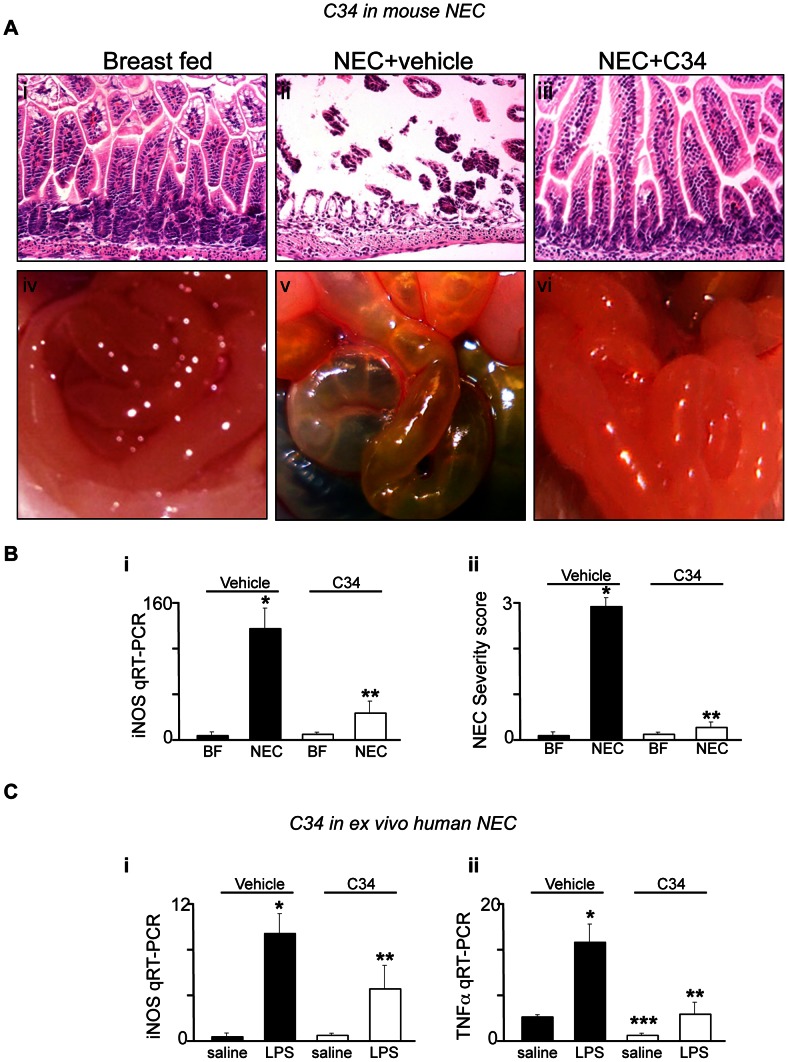
C34 attenuates intestinal inflammation in mouse and human NEC. **A**. Representative photomicrographs (**i–iii**) and gross images (**iv–vi**) of the ileum from neonatal mice that were either breast fed (**i**, **iv**), induced to develop NEC along with vehicle (**ii**, **v**), or induced to develop NEC in the presence of C34 (1 mg/kg daily) as described in Methods. **B**: qRT-PCR showing the expression of iNOS in the intestinal mucosa (**i**) and NEC severity score (**ii**) of newborn mice that were either breast fed (“BF”) or induced to develop NEC in the absence (black bars) or presence (white bars) of C34 (1 mg/kg/day). Shown are mean±SEM. *p<0.05 vehicle NEC vs breast fed; **p<0.05 NEC+34 vs NEC+vehicle. Representative of 5 separate experiments with over 5 neonatal mice per group. **C:** qRT-PCR showing expression of iNOS (**i**) and TNFα (**ii**) in the resected ileal tissue from neonates with NEC that was subsequently treated with saline or LPS in the presence of vehicle or C34 for 3 hours. Shown is mean±SEM from 3 separate specimens; *p<0.05 LPS vs saline; ***p<0.05 LPS+vehicle vs C34+ saline; **p<0.05 LPS+vehicle versus LPS+C34. The y axes in panels B and C indicate the fold increase of the indicated gene relative to the housekeeping gene GAPDH.

NEC is the leading cause of death from gastrointestinal disease in premature infants, and is characterized by a marked increase in acute inflammation of the intestinal mucosa that leads to epithelial cell death and systemic sepsis [24]. Despite advances in neonatal care, overall survival has remained unchanged in the past two decades, in large part due to a lack of specific therapies for this devastating disease [25]. We [26] and others [27] recently determined that the development of NEC in mice, rats and humans reflects increased TLR4 signaling in the premature host, as exaggerated TLR4 signaling within the gut leads to increased enterocyte apoptosis and elevated pro-inflammatory cytokine release [3]. In order to test directly whether C34 could attenuate NEC severity, we administered by oral gavage C34 in a well validated model of experimental NEC in mice that consisted of four days of formula by gavage as well as twice daily hypoxic treatments [25]. NEC severity was measured by the degree of inflammation within the intestinal mucosa and maintenance of the mucosal architecture according to a validated scoring system, as well as the extent of expression of the pro-inflammatory cytokine iNOS within the intestinal mucosa [25]. Mice with NEC that were administered saline alone demonstrated marked intestinal inflammation and disruption of the intestinal mucosa (**Figure 4A** **ii** versus **i**), necrosis of the small intestine (**Figure 4A** **iv** versus **v**), increased expression of the pro-inflammatory cytokine iNOS in the intestinal mucosa (**Figure 4B** **i**), and an increase in the NEC severity score as we had previously validated [18] (**Figure 4B** **ii)**. Importantly, mice that received C34 demonstrated a marked preservation of the intestinal mucosa (**Figure 4A** **iii** versus **ii**), a reduction in the appearance of gross disease (**Figure 4A** **v** versus **vi**), a reduction in the extent of pro-inflammatory iNOS expression (**Figure 4B** **i**) and a reduction in NEC severity score (**Figure 4B** **ii**). Taken together, these findings demonstrate that the novel TLR4 inhibitor, C34, attenuates NEC severity.

### C34 also Inhibits TLR4 Signaling in Human Tissue ex vivo Obtained from Infants with NEC

In a final series of studies, we sought to evaluate whether C34 could inhibit TLR4 signaling in human intestinal tissue in patients with NEC. To do so, we obtained freshly isolated small intestine from human infants that had undergone surgical resection for NEC, which necessarily requires the removal of a small amount of inflamed yet viable tissue. After gross review by the pediatric duty pathologist, the viable tissue was randomly sectioned into multiple pieces, and treated with either LPS, LPS after C34 pre-treatment, and C34 alone. As shown in **Figure 4C** **i–ii**, LPS significantly increased the expression of iNOS (**Figure 4C** **i**) and TNFα (**Figure 4C** **ii**), but this effect was reversed by pre-treatment with C34. Of note, tissue that was treated with C34 alone showed a marked decrease in TNFα expression without LPS treatment, suggesting that C34 reduced ongoing inflammation in the resected tissue. Taken together, our findings show that C34 decreases the extent of LPS signaling in human tissue, and raises the exciting possibility that C34 represents a new therapeutic option in diseases associated with exaggerated TLR4 signaling, such as NEC.

## Discussion

We report the results of a computational similarity search-assisted drug discovery approach for the identification of a new class of small organic molecules with shared structural identities based on E5564. Through a series of confirmatory studies, we established that our lead compound C34 inhibits TLR4 *in vitro* in macrophages and enterocytes, and *in vivo* in experimental models of endotoxemia and NEC. In addition, C34 reduced the degree of LPS signaling in human tissue that was freshly removed from infants with NEC. Based upon our current findings, we have identified a new class of small molecules with therapeutic potential in diseases that are characterized by exaggerated TLR4 signaling.

C34, which is a novel inhibitor of TLR4 *in vitro* and *in vivo*, has the molecular formula of C_17_H_27_NO_9_, a molecular weight of 389.40, and a computed docking arrangement shown in Figure 2. While we now describe the first report of this particular molecule showing anti-TLR4 effects, the current findings are consistent with other studies that have suggested a role for 2-amino-2-deoxyglycosides as anti-inflammatory agents. Inhibitor E5564, which was used as a reference structure for its ability to bind to TLR4 in the initial *in silico* screen, has a 2-acylamidopyranoside substructure. Furthermore, the *N*-acetyl-glucosamine scaffold has known anti-inflammatory effects in a variety of cells, including retinal pigment epithelial cells [28], endothelial cells [29], mast cells [30] and peritoneal mesothelial cells [31], providing further evidence for the role for this class of molecules as TLR4 inhibitors. Interestingly, Lee and colleagues also recently showed that a novel aminosaccharide has anti-TLR effects in macrophages *in vitro* [32]. Based on these findings, we submit that per-acylated 2-aminopyranoses with alkyl side chains at the anomeric carbon, in particular those with similarity to our lead compound, C34, have the potential to elicit broad and beneficial clinical effects in diseases characterized by TLR4 hyperactivation. Given that the development of necrotizing enterocolitis occurs almost exclusively in premature infants after they have received oral feeds, and given our findings that the development of NEC reflects increased signaling via TLR4 in the intestinal epithelium of the premature gut [3], [16], [18], [26], [33], it is exciting to speculate that a feeding regimen which contains a TLR4 inhibitor such as C34 may be of benefit in the prevention of NEC in this population.

An exciting and rather serendipitous discovery in the current work links our identification of this aminoglycoside scaffold (i.e., C34 and the closely related β-anomeric cyclohexyl analog C35) to the treatment of experimental NEC, a disease that we and others have shown to be mediated via TLR4 activation in mice and humans [26], [27]. Although a cure for NEC remains elusive, there is consensus that the administration of breast milk is the most effective preventive strategy [26], [27]. While the precise mechanisms to explain the protective effects of breast milk remain unknown, an emerging body of literature has determined that breast milk-derived oligosaccharides exert anti-inflammatory properties, and that these molecules may be responsible in part for the protective effects of breast milk in NEC. For instance, human milk oligosaccharides reduce leukocyte adhesion to endothelial cells [34], reduce platelet-leukocyte adhesion [35], alter the composition of the microbiome [36], and alter the degree of adherence of certain microbes to the surface of the intestinal epithelium [37]. The specific mechanisms by which human milk oligosaccharides exert these protective effects remain incompletely understood. Given our finding that simple aminomonosaccharides serve as TLR4 inhibitors, it is possible that human milk oligosaccharides convey their anti-inflammatory function in part through a previously unrecognized inhibitory effect on TLR4 signaling.

In summary, we have identified a new class of small molecule inhibitors of TLR4, which show substantial benefits in models of experimental NEC. These findings suggest a viable approach for the development of novel therapies for a variety of infectious and inflammatory diseases in which TLR4 signaling plays a critical role.
